# Supplementary material for: Diagnosis and management of endometrial hyperplasia: A UK national audit of adherence to national guidance 2012–2020
Source: PLoS Med. 2024 Feb 29;21(2):e1004346. doi: 10.1371/journal.pmed.1004346 (PMC10903889; doi:10.1371/journal.pmed.1004346)
Supplement: S1 Text — (DOCX) [file pmed.1004346.s002.docx]

**S1 Text. UKARCOG Working Group Authors**

Islam Abaza, Mahmoud Abdelghaffar, Charlotte Atkinson, Kula Ayad, Liam J Beamer, Catherine Bear, Deborah Blower, Vijna Boodhoo, Sophie Bracke, Christi AM Brasted, Emily Brook, Kieran Broome, Alix Bullock, Hannah Burkey, Andy Catton, Uzochukwu Chukwujama, Ewa Ciolak, Amy Cleese, Sophie Collins, Natasha Craig, Annabel Creeth, Natalie Croghan, Anusha D’Sa, Ananya Das, Hanna Davies, Jennifer Davies, Melin Dokmeci, Louise Dunphy, Jade Edwards, Mohamed El-Sherbiny, Babjide Erinle, Rinata Farah, Hira Fatima, Galine Fattal, Teresa Fung, Laura Gooch, Rosie Grainger, Lucy Hanson, Francesca Hogg, Tom Hussey, Geetu Jethwani, Sarah Jung, Maria K Kaloudi, Anthie Karavaggelis, Shilpaja Karpate, Alex J Kermack, Tabassum Khan, Fatima Khattak, Shen C Khaw, Emily Knight, Snehal Kolluru, Sujatha Kumari, Aarti Lakhiani, Natalie Macleod, Caroline MacMahon, Misbah Malik, Alison Martin, Ashwini Maudhoo, Bethaney Mayhew, Claire McCormack, Sherif Mito, Fatema Mohammed Ali, Alison Montgomery, Roberta Morris, Sughashini Murugesu, Emma Nash, Adam Naskretski, Mohamed AN Nasr, Simrit Nijjar, Emer O’Donnell, Tejumole Olaoye, Gemma Owens, Victoria Pereira, Zahra Pervaiz, Mark Pickering, Hannah Pierce, Charlotte Plant, Shahin Qadri, Olivia Raglan, Meera Ramcharn, Tahira Rashid, Meenachi Rathnavelu, Abiman Ravishanker, Jonathan Riley, Nicola Roberts, Peter Sanderson, Sophie Sharples, Vidya Shyam-Sundar, Mona Soleymani, Christos Spyroulis, Sophie Stezaker, Alice Stickland, Linden J Stocker, Yong Shen Tan, Dilruba K Toma, Prema Ulganathan, Shaheen Uqaili, Arjun Vora, Sarah Woldman, Rekha Wuntakal, Charlotte Wyeth, Sihao Zhao
