# Supplementary material for: Diagnosis and management of endometrial hyperplasia: A UK national audit of adherence to national guidance 2012–2020
Source: PLoS Med. 2024 Feb 29;21(2):e1004346. doi: 10.1371/journal.pmed.1004346 (PMC10903889; doi:10.1371/journal.pmed.1004346)
Supplement: S1 Table — (DOCX) [file pmed.1004346.s003.docx]

**S1 Table.** Participating Hospitals

| **Region** | **Trust** | **Hospital** |
| --- | --- | --- |
| East of England | Milton Keynes University Hospital NHS Foundation Trust | Milton Keynes University Hospital, Milton Keynes |
| London | Royal Free London NHS Foundation Trust | Barnet Hospital, London |
|  | Barking, Havering and Redbridge University Hospitals NHS Trust | Queens Hospital, London |
|  | Chelsea and Westminster Hospital NHS Foundation Trust | West Middlesex University Hospital, London; Chelsea and Westminster Hospital, London |
|  | Imperial College Healthcare NHS Trust | Queen Charlotte's and Chelsea Hospital, London; St Mary's Hospital, London |
|  | Lewisham and Greenwich NHS Trust | University Hospital Lewisham, London |
|  | The Hillingdon Hospitals NHS Foundation Trust | Hillingdon Hospital, London |
| Midlands | University Hospitals Coventry and Warwickshire NHS Trust | University Hospital Coventry, Coventry |
|  | Birmingham Women's and Children's NHS Foundation Trust | Birmingham Women's Hospital, Birmingham |
|  | George Eliot Hospital NHS Trust | George Eliot Hospital, Nuneaton |
|  | Royal Wolverhampton NHS Trust | New Cross Hospital, Wolverhampton |
|  | Sandwell and West Birmingham Hospitals NHS Trust | Sandwell General Hospital and City Hospital, Birmingham |
|  | Shrewsbury and Telford NHS Trust | The Princess Royal Hospital, Telford and The Royal Shrewsbury Hospital, Shrewsbury |
|  | South Warwickshire NHS Foundation Trust | Warwick Hospital, Warwick |
|  | University Hospitals Birmingham NHS Foundation Trust | Good Hope Hospital and Heartlands Hospital, Birmingham |
|  | University Hospitals North Midlands | Royal Stoke University Hospital, Stoke-On-Trent |
|  | Walsall Healthcare NHS Trust | Walsall Manor Hospital, Walsall |
|  | Worcestershire Acute Hospitals NHS Trust | Worcestershire Royal Hospital, Worcester |
| North East and Yorkshire | Newcastle Hospitals NHS Foundation Trust | Royal Victoria Infirmary, Newcastle |
|  | Airedale NHS Foundation Trust | Airedale General Hospital |
|  | Calderdale and Huddersfield NHS Foundation Trust | Royal Calderdale Hospital, Halifax and Huddersfield Royal Infirmary, Huddersfield |
|  | County Durham and Darlington NHS Foundation Trust | Darlington Memorial Hospital, Darlington; University Hospital of North Durham, Durham |
|  | Hull University Teaching Hospitals NHS Trust | Hull Royal Infirmary and Castle Hill Hospital, Hull |
|  | Mid Yorkshire Hospitals NHS Trust | Pinderfields Hospital, Dewsbury and District Hospital and Pontefract Hospital, Yorkshire |
|  | North Cumbria Integrated Care NHS Foundation Trust | Cumberland Infirmary |
|  | South Tees Hospitals NHS Foundation Trust | James Cook University Hospital, Middlesborough |
|  | South Tyneside and Sunderland NHS Foundation Trust | Sunderland Royal Hospital, Sunderland |
|  | York and Scarborough Teaching Hospitals NHS Foundation Trust | York Hospital, York and Scarborough Hospital, Scarborough |
| North West | Liverpool Women's NHS Foundation Trust | Liverpool Women's Hospital, Liverpool |
|  | Bolton NHS Foundation Trust | Royal Bolton Hospital, Bolton |
|  | Countess of Chester Hospital NHS Foundation Trust | Countess of Chester Hospital, Chester |
|  | East Lancashire Hospitals NHS Trust | Burnley General Teaching Hospital, Burnley |
|  | Lancashire Teaching Hospitals NHS Foundation Trust | Royal Preston Hospital, Preston |
|  | Manchester University NHS Foundation Trust | Saint Mary's Hospital, Manchester |
|  | Mid Cheshire Hospitals NHS Foundation Trust | Leighton Hospital, Crewe |
|  | Northern Care Alliance NHS Foundation Trust | Royal Oldham Hospital, Oldham |
|  | Southport and Ormskirk Hospital NHS Trust | Ormskirk District General Hospital, Ormskirk |
|  | Tameside and Glossop Integrated Care NHS Foundation Trust | Tameside Hospital, Tameside |
|  | University Hospitals of Morecambe Bay NHS Foundation Trust | Royal Lancaster Infimary, Lancaster |
|  | Warrington and Halton Teaching Hospitals NHS Foundation Trust | Warrington Hospital, Warrington |
|  | Wirral University Teaching Hospital NHS Foundation Trust | Arrowe Park Hospital, The Wirral |
|  | Wrightington, Wigan and Leigh Teaching Hospitals NHS Foundation Trust | Royal Albert Edward Infimary, Wigan |
| South East | Ashford and St Peter's Hospitals NHS Foundation Trust | St Peter's Hospital, Chertsey |
|  | Buckinghamshire Healthcare NHS Trust | Stoke Mandeville Hospital, Aylesbury |
|  | East Kent Hospitals University NHS Foundation Trust | Queen Elizabeth The Queen Mother Hospital, Margate; William Harvey Hospital, Ashford |
|  | Frimley Health NHS Foundation Trust | Wexham Park Hospital, Slough |
|  | Hampshire Hospitals NHS Foundation Trust | Royal Hampshire County Hospital, Winchester; Basingstoke and North Hampshire Hospital, Basingstoke |
|  | Medway NHS Foundation Trust | Medway Maritime Hospital |
|  | Oxford University Hospitals NHS Foundation Trust | John Radcliffe Hospital, Oxford |
|  | Portsmouth Hospitals NHS Trust | Queen Alexandra Hospital, Portsmouth |
|  | Royal Berkshire NHS Foundation Trust | Royal Berkshire Hospital, Reading |
|  | Surrey and Sussex Healthcare NHS Trust | East Surrey Hospital, Redhill |
|  | University Hospital Southampton NHS Foundation Trust | Princess Anne Hospital, Southampton |
| South West | Dorset County Hospital NHS Foundation Trust | Dorset County Hospital, Dorchester |
|  | Salisbury NHS Foundation Trust | Salisbury Hospital, Salisbury |
|  | University Hospitals Dorset NHS Foundation Trust | Poole Hospital, Poole |
| Scotland | NHS Greater Glasgow and Clyde | Queen Elizabeth University Hospital, Glasgow; Princess Royal Maternity, Glasgow |
|  | NHS Tayside | Ninewells Hospital, Dundee |
| Wales | Cwm Taf Morgannwg University Health Board | Princess of Wales Hospital, Bridgend |
|  | Aneurin Bevan University Health Board | Royal Gwent Hospital, Newport |
|  | Betsi Cadwaladr University Health Board | Wrexham Maelor Hospital, Wrexham; Ysbyty Gwynedd, Bangor |
|  | Hywel Dda University Health Board | Glangwili Hospital, Carmarthen |
|  | Swansea Bay University Health Board | Singleton Hospital, Swansea |
