# Supplementary material for: Diagnosis and management of endometrial hyperplasia: A UK national audit of adherence to national guidance 2012–2020
Source: PLoS Med. 2024 Feb 29;21(2):e1004346. doi: 10.1371/journal.pmed.1004346 (PMC10903889; doi:10.1371/journal.pmed.1004346)
Supplement: S2 Table — (DOCX) [file pmed.1004346.s004.docx]

**S2 Table. Characteristics of patients with non-atypical or atypical endometrial hyperplasia diagnosed during 2020**

|  | | NEH | | | | AEH | |
| --- | --- | --- | --- | --- | --- | --- | --- |
|  | | | 85 | | | 141 | |
|  | | | **N** | **%** | | **N** | **%** |
| Age, mean years (SD) | 54 (12) | | |  | 60 (11) | |  |
| Missing | 0 | | |  | 2 | |  |
|  |  | | |  |  | |  |
| Body mass index |  | | |  |  | |  |
| <25 | 11 | | | 13 | 11 | | 7.8 |
| 25-29 | 14 | | | 16 | 9 | | 6.4 |
| 30-34 | 5 | | | 5.9 | 8 | | 5.7 |
| 35-39 | 8 | | | 9.4 | 23 | | 16 |
| ≥40 | 15 | | | 18 | 32 | | 23 |
| Missing | 32 | | | 38 | 58 | | 41 |
|  |  | | |  |  | |  |
| Diabetes | 14 | | | 16 | 18 | | 13 |
| PCOS | 4 | | | 4.7 | 3 | | 2.1 |
| Hypertension | 27 | | | 32 | 54 | | 38 |
|  |  | | |  |  | |  |
| Smoking |  | | |  |  | |  |
| Never smoked | 52 | | | 61 | 108 | | 77 |
| Ex-smoker | 6 | | | 7.0 | 6 | | 4.3 |
| Current/recently stopped | 4 | | | 13 | 4 | | 2.8 |
| Missing | 23 | | | 19 | 23 | | 16 |
|  |  | | |  |  | |  |
| Any HRT use | 11 | | | 13 | 8 | | 5.7 |
| Any tamoxifen use | 4 | | | 4.7 | 4 | | 2.8 |
|  |  | | |  |  | |  |
| Previous births |  | | |  |  | |  |
| 0 | 20 | | | 24 | 21 | | 15 |
| 1 | 9 | | | 11 | 17 | | 12 |
| 2 | 23 | | | 27 | 46 | | 33 |
| ≥3 | 23 | | | 27 | 18 | | 13 |
| Missing | 10 | | | 12 | 39 | | 28 |
|  | | |  |  |  | |  |
| Presenting complaint | | |  |  |  | |  |
| Postmenopausal bleeding | | | 54 | 64 | 116 | | 82 |
| Heavy menstrual bleeding | | | 17 | 20 | 12 | | 8.5 |
| Intermenstrual bleeding | | | 9 | 11 | 8 | | 5.7 |
| Incidental finding | | | 2 | 2.4 | 3 | | 2.1 |
| Subfertility | | | 1 | 1.2 | 1 | | 0.71 |
| Post-coital bleeding | | | 1 | 1.2 | 1 | | 0.71 |
| NEH Non-atypical endometrial hyperplasia, AEH Atypical endometrial hyperplasia PCOS Polycystic ovary syndrome, HRT Hormone replacement therapy.  Proportions may not sum to 100% due to rounding. | | | | | | | |
