# Supplementary material for: Diagnosis and management of endometrial hyperplasia: A UK national audit of adherence to national guidance 2012–2020
Source: PLoS Med. 2024 Feb 29;21(2):e1004346. doi: 10.1371/journal.pmed.1004346 (PMC10903889; doi:10.1371/journal.pmed.1004346)
Supplement: S3 Table — (DOCX) [file pmed.1004346.s005.docx]

**S3 Table. First-line treatment of patients who were diagnosed with non-atypical or atypical endometrial hyperplasia during 2020 and comparison with a pre-pandemic baseline (2016-19)**

| NEH | N | % (95% CI) | RR (95% CI) | *p-value* |
| --- | --- | --- | --- | --- |
| First-line treatment |  |  |  |  |
| None offered or declined | 3 | 3.6 (0.98-12) | 1.22 (0.35-4.27) | *0.76* |
| Weight loss (any) | 3 | 3.6 (1.1-11) | 0.64 (0.18-2.29) | *0.49* |
| Further investigation >42 days | 8 | 9.5 (4.4-20) | 0.83 (0.39-1.75) | *0.62* |
|  |  |  |  |  |
| Any continuous progesterone^a^ | 62 | 73 (55-85) | 1.07 (0.82-1.39) | *0.63* |
| Intrauterine progesterone | 46 | 54 (36-70) | 1.06 (0.77-1.46) | *0.73* |
| Oral progesterone | 14 | 17 (9.2-28) | 0.88 (0.50-1.55) | *0.65* |
|  |  |  |  |  |
| Endometrial ablation | 0 | - | - | - |
| Hysterectomy | 11 | 13 (6.6-24) | 1.15 (0.60-2.21) | *0.68* |
|  |  |  |  |  |
| AEH |  | |  | |
| First-line treatment | 59 | 42 (31-54) | 1.62 (1.18-2.21) | *0.003* |
| None offered or declined | 3 | 2.1 (0.60-7.3) | 1.29 (0.35-4.78 | *0.70* |
| Weight loss (any) | 1 | 0.71 (0.080-6.0) | 0.29 (0.036-2.40) | *0.25* |
| Further investigation >42 days | 9 | 6.4 (2.8-14) | 1.46 (0.63-3.38) | *0.38* |
|  |  |  |  |  |
| Any continuous progesterone^a^ | 59 | 58 (46-69) | 1.62 (1.18-2.21) | 0.003 |
| Intrauterine progesterone | 47 | 33 (26-42) | 1.60 (1.13-2.28) | *0.009* |
| Oral progesterone | 13 | 9.2 (5.0-16) | 1.55 (0.82-2.94) | *0.18* |
|  |  |  |  |  |
| Endometrial ablation | 0 | - | - | - |
| Hysterectomy | 74 | 52 (42-63) | 0.78 (0.61-0.99) | *0.042* |
| NEH Non-atypical endometrial hyperplasia, AEH Atypical endometrial hyperplasia Proportions are with clustered standard errors. | | | | |
| First-line treatment includes all treatments following first biopsy and following further investigations or biopsies within 42 days. | | | | |
| ^a^ Intrauterine, oral, or intramuscular | | | | |
| Rate ratios compare women who received a diagnosis of EH in 2020 with those in 2016-2019 | | | | |
