# Supplementary material for: Diagnosis and management of endometrial hyperplasia: A UK national audit of adherence to national guidance 2012–2020
Source: PLoS Med. 2024 Feb 29;21(2):e1004346. doi: 10.1371/journal.pmed.1004346 (PMC10903889; doi:10.1371/journal.pmed.1004346)
Supplement: S4 Table — (DOCX) [file pmed.1004346.s006.docx]

**S4 Table. Proportion of patients with AEH who underwent first-line hysterectomy and unadjusted and adjusted rate ratios for first-line hysterectomy according to their characteristics**

|  | **First-line hysterectomy** | | | |  |  |  |  |
| --- | --- | --- | --- | --- | --- | --- | --- | --- |
|  | **Pre-guidance** | | **Post-guidance** | |  |  |  |  |
|  | N | % | N | % | RR (95% CI) | *p-value* | aRR (95% CI) | *p-value* |
|  | 453 |  | 569 |  |  |  |  |  |
| **Age, years** |  |  |  |  |  |  |  |  |
| <40 | 11 | 22 (11-40) | 15 | 20 (12-31) | 0.28 (0.19-0.41) | *<0.001* | 0.23 (0.12-0.43) | *<0.001* |
| 40-49 | 70 | 71 (60-79) | 88 | 71 (61-79) | 0.96 (0.80-1.16) | *0.68* | 0.98 (0.72-1.33) | *0.90* |
| 50-59 | 134 | 72 (57-83) | 216 | 75 (68-81) | 1.00 (ref) | *-* | 1.00 (ref) | *-* |
| 60-69 | 139 | 67 (57-76) | 162 | 73 (67-79) | 0.96 (0.82-1.12) | *0.57* | 1.02 (0.81-1.28) | *0.87* |
| ≥70 | 91 | 81 (73-87) | 84 | 65 (58-71) | 0.98 (0.82-1.17) | *0.81* | 1.01 (0.78-1.32) | *0.91* |
| Missing | 8 | 67 (31-90) | 4 | 100 | - |  | - |  |
|  |  |  |  |  |  |  |  |  |
| **Body mass index, kg/m^2^** | | |  |  |  |  |  |  |
| <25 | 32 | 53 (26-79) | 74 | 80 (69-88) | 1.00 (ref) | *-* | 1.00 (ref) | *-* |
| 25-29 | 61 | 73 (48-89) | 80 | 76 (65-84) | 1.09 (0.85-1.40) | *0.50* | 1.08 (0.82-1.44) | *0.58* |
| 30-34 | 67 | 76 (67-84) | 107 | 74 (65-81) | 1.08 (0.85-1.38) | *0.51* | 1.18 (0.90-1.55) | *0.24* |
| 35-39 | 73 | 79 (67-88) | 92 | 70 (58-80) | 1.07 (0.84-1.37) | *0.56* | 1.16 (0.87-1.55) | *0.31* |
| ≥40 | 71 | 50 (39-60) | 108 | 51 (43-60) | 0.74 (0.58-0.94) | *0.014* | 0.76 (0.57-1.03) | *0.075* |
| Missing | 149 | 74 (68-79) | 108 | 68 (58-77) | - |  |  |  |
|  |  |  |  |  |  |  |  |  |
| Diabetes | 74 | 61 (47-73) | 74 | 51 (41-61) | 0.83 (0.69-0.99) | *0.034* | 0.84 (0.66-1.08) | *0.18* |
| PCOS | 8 | 40 (22-61) | 10 | 38 (24-53) | 0.53 (0.34-0.85) | *0.009* | 1.04 (0.58-1.79) | *0.94* |
| Hypertension | 165 | 67 (56-77) | 195 | 59 (53-66) | 0.95 (0.83-1.08) | *0.43* | 0.92 (0.76-1.13) | *0.44* |
|  |  |  |  |  |  |  |  |  |
| **Smoking** |  |  |  |  |  |  |  |  |
| Never smoked | 252 | 64 (51-75) | 398 | 69 (64-73) | 1.00 (ref) | *-* | 1.00 (ref) | *-* |
| Ex-smoker | 30 | 77 (60-88) | 32 | 62 (46-75) | 1.02 (0.79-1.32) | *0.89* | 1.03 (0.76-1.39) | *0.84* |
| Current/ recently stopped | 32 | 67 (51-80) | 39 | 67 (51-81) | 1.01 (0.79-1.29) | *0.93* | 1.04 (0.78-1.40) | *0.78* |
| Missing | 139 | 75 (68-80) | 100 | 64 (55-72) | - |  | - |  |
|  |  |  |  |  |  |  |  |  |
| Any HRT use | 30 | 79 (60-90) | 41 | 85 (72-92) | 1.32 (1.04-1.68) | *0.023* | 1.20 (0.87-1.67) | *0.27* |
| Any tamoxifen use | 19 | 79 (51-93) | 34 | 86 (71-94) | 1.24 (0.94-1.63) | *0.13* | 1.05 (0.72-1.52) | *0.81* |
|  |  |  |  |  |  |  |  |  |
| **Previous births** |  |  |  |  |  |  |  |  |
| 0 | 69 | 53 (42-63) | 92 | 56 (47-64) | 0.75 (0.62-0.91) | *0.004* | 1.00 (0.79-1.27) | *0.98* |
| 1 | 46 | 69 (53-81) | 83 | 69 (60-76) | 0.95 (0.77-1.17) | *0.60* | 1.06 (0.83-1.37 | *0.63* |
| 2 | 106 | 71 (52-85) | 174 | 73 (65-79) | - | *-* | - | *-* |
| ≥3 | 81 | 68 (51-81) | 113 | 67 (57-75) | 0.94 (0.78-1.13) | *0.51* | 1.01 (0.82-1.26) | *0.91* |
| Missing | 151 | 75 (67-82) | 107 | 72 (65-79) | - |  | - |  |
|  |  |  |  |  |  |  |  |  |
| **Presenting complaint** |  |  |  |  |  |  |  |  |
| Postmenopausal bleeding | 306 | 69 (56-80) | 405 | 74 (69-78) | 1.19 (1.04-1.36) | *0.010* | 0.99 (0.68-1.43) | *0.95* |
| Heavy menstrual bleeding | 56 | 67 (54-78) | 65 | 53 (43-62) | 0.85 (0.71-1.03) | *0.092* | 1.00 (0.68-1.46) | *0.98* |
| Intermenstrual bleeding | 20 | 59 (45-71) | 41 | 59 (44-71) | 0.86 (0.66-1.11) | *0.25* | 1.06 (0.71-1.58) | *0.77* |
| Incidental finding | 27 | 71 (53-84) | 37 | 64 (53-74) | 0.98 (0.76-1.27) | *0.91* | 0.97 (0.60-1.58) | *0.9* |
| Subfertility | 0 | - | 2 | 20 (3.5-63) | 0.13 (0.032-0.51) | *0.004* | 0.62 (0.14-2.71) | *0.53* |
| Post-coital bleeding | 3 | 75 (4.1-100) | 10 | 56 (29-79) | 0.87 (0.51-1.51) | *0.62* | 0.94 (0.45-1.90) | *0.83* |
| AEH Atypical endometrial hyperplasia, PCOS Polycystic ovary syndrome, HRT Hormone replacement therapy | | | | | | | | |
| RR represents unadjusted rate ratios; aRR represents rate ratios from the full mutually-adjusted model (all risk factors shown are included) | | | | | | | | |
