# Supplementary figures and images for: Diagnosis and management of endometrial hyperplasia: A UK national audit of adherence to national guidance 2012–2020
Source: PLoS Med. 2024 Feb 29;21(2):e1004346. doi: 10.1371/journal.pmed.1004346 (PMC10903889; doi:10.1371/journal.pmed.1004346)

S1 Fig

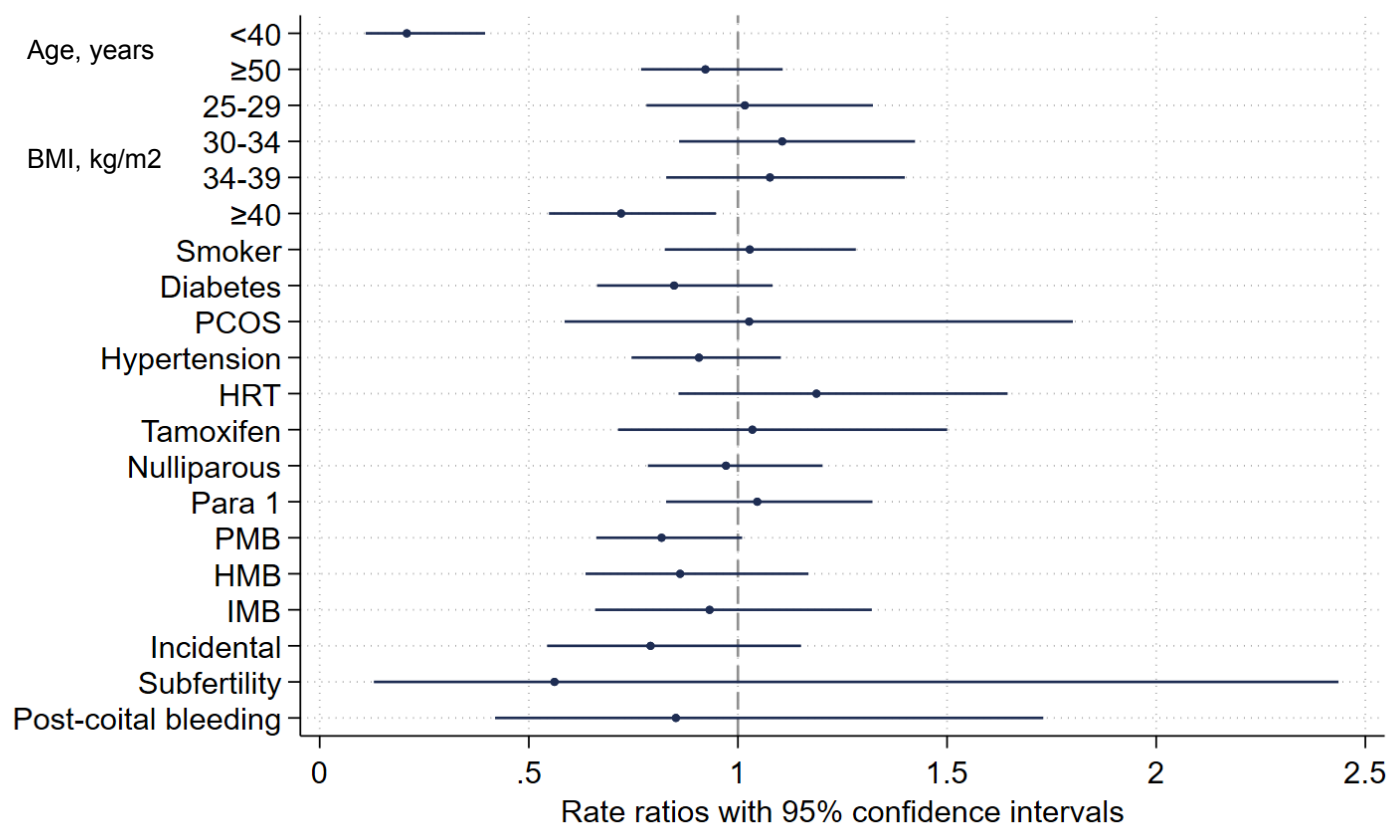

Supplement: S1 Fig — Rate ratios with 95% confidence intervals for first-line hysterectomy for the mutually adjusted risk-factors. The baseline group for age is 40–49 years, for BMI is <25, and for parity is para 2 or greater. Some levels of age, BMI, and parity were combined where these estimates were near identical. (PDF) [file pmed.1004346.s007.pdf]
