# Supplementary material for: Diagnosis and management of endometrial hyperplasia: A UK national audit of adherence to national guidance 2012–2020
Source: PLoS Med. 2024 Feb 29;21(2):e1004346. doi: 10.1371/journal.pmed.1004346 (PMC10903889; doi:10.1371/journal.pmed.1004346)
Supplement: Attachment — Submitted filename: Response to reviewers_re2.pdf [file pmed.1004346.s009.pdf]

Response to review feedback

Dear reviewers,

Thank you for your further comments which we have responded to below.

Comments from the Editors:

Thank you for carefully revising your manuscript, and we thank the authors for their responses.

We like to suggest you include "2012-20" somewhere in your title.

This has now been included.

At line 50-51, please add when was the guideline introduced

We have included this where omitted from the author summary.

The abstract should detail the number of events of events for outcomes (as opposed to only percentages).

Also in the abstract, please include the absolute risk(s) of relevant outcomes, not just relative risks or correlation coefficients. (example for absolute risks: PMID: 28399126).

The abstract has been updated with the counts where omitted, as well as the absolute differences where we had previously reported the relative measures.

In the Discussion section, we also ask that you discuss in detail the absolute risk as compared to the relative risk presented regarding the implications and how these findings should be interpreted by the public/individual. We feel that this is extremely important with this type of study, so that readers can appropriately interpret the risk data. Please also provide the absolute risk data as an SI (or where you feel appropriate)

Thank you for highlighting this issue. We have included the absolute differences in the principle findings and have incorporated ARDs 'absolute rate differences' (in keeping with the terminology for RRs) into the tables and results.

In line with the statistical reviewer we invite you to review the risk reporting and take care to not overinflate interpretable risk.

Apologies for this error, which has been corrected.

If you have any question about the above, please do hesitate to contact me directly at kjanin@plos.org

----

Comments from Reviewers:

Reviewer #1: The authors have been responsive to the reviewers' concerns. Multiple components of the methods and results are clearer and more fully described.

Reviewer #2: There is one typo-graphic error. Please see page 9 line 274 for clean copy.

Data were collected on age (<40, 40-49, 50-59, 60-69, ≥40 years);

>=40 will be >=70

Thank you for highlighting this error, which we have corrected.

Reviewer #3: Alex McConnachie, Statistical Review

I thank the authors for their responses to my previous comments, all of which are satisfactory.

I have just a few very minor corrections to suggest:

- *Line 332: should this be "…women with AEH"?*
- *Line 337: "decreased by 70%" is not accurate, given the RR of 0.36*
- *Line 363: should this be "…[46, 66%]) post-guidance."?*
- *Line 405: should be "decreased by 28%"*

Thank you for highlighting these errors, and apologies - these have been addressed.
