# Supplementary material for: Diagnosis and management of endometrial hyperplasia: A UK national audit of adherence to national guidance 2012–2020
Source: PLoS Med. 2024 Feb 29;21(2):e1004346. doi: 10.1371/journal.pmed.1004346 (PMC10903889; doi:10.1371/journal.pmed.1004346)
Supplement: Attachment — Submitted filename: Response to reviewers EH_re.docx [file pmed.1004346.s008.docx]

| **Requests from the editors:** | | | |  |
| --- | --- | --- | --- | --- |
| **No.** | **Area** | **Comment** | **Response** | **Line ref** |
| 1 | General | Please organise your manuscript as follows and use the following headings: Abstract, Introduction, Methods, Results, Discussion, Conclusion (1 paragraph) | Thank you, we have changed the manuscript accordingly. |  |
| 2 | General | Please provide 95% CIs and p values for all results were appropriate (including the abstract), check and amend throughout. We suggest reporting statistical information in the following format: ‘x’; (95% CI [‘y’,’ z’] p value) For p values, please report as p<0.001 and where higher as 'p=0.002'. | We have included the previously omitted p values for risk ratios and for all references to the estimates throughout the text. We have reformatted the estimates and 95% confidence intervals accordingly. |  |
| 3 | General | Please add the statistical method used to your method section. | Apologies for this omitted detail. We have updated the methods including the new analyses conducted in response to the feedback. |  |
| 4 | General | Please define all abbreviations for the reader at first use | We have updated any omitted initialisms, thank you. |  |
| 5 | General | For Figure and Tables (including in SI) , please redefine all abbreviations in the legends. | We have repeated all initialisms in all figures and tables, thank you. |  |
| 6 | General | For in-text reference, citations are placed within square parentheses and should precede punctuation. Please check and amend throughout. | This has been amended. |  |
| 7 | General | Please use line numbers for ease of referencing during the revision process. | Line numbers have been included in the manuscript. |  |
| 8 | Study design | For all observational studies (including audits), in the manuscript text, please indicate: (1) the specific hypotheses you intended to test, (2) the analytical methods by which you planned to test them, (3) the analyses you actually performed, and (4) when reported analyses differ from those that were planned, transparent explanations for differences that affect the reliability of the study's results. If a reported analysis was performed based on an interesting but unanticipated pattern in the data, please be clear that the analysis was data-driven. | We have added the null hypothesis to the objectives paragraph. We have updated the methods with the statistical analyses section, and have explicitly clarified any post-hoc analyses at the end of the statistical analyses section. | 262 |
| 9 | Study design | Please ensure that the study is reported according to the STROBE guideline, and include the completed STROBE checklist as Supporting Information. Please add the following statement, or similar, to the Methods: "This study is reported as per the Strengthening the Reporting of Observational Studies in Epidemiology (STROBE) guideline (S1 Checklist)."  The STROBE guideline can be found here: [http://www.equator-network.org/reporting-guidelines/strobe/](https://eur01.safelinks.protection.outlook.com/?url=http%3A%2F%2Fwww.equator-network.org%2Freporting-guidelines%2Fstrobe%2F&data=05%7C01%7C%7Cfee979e3ac594d175ebe08dbcbcbf1f8%7C09bacfbd47ef446592653546f2eaf6bc%7C0%7C0%7C638327849040446571%7CUnknown%7CTWFpbGZsb3d8eyJWIjoiMC4wLjAwMDAiLCJQIjoiV2luMzIiLCJBTiI6Ik1haWwiLCJXVCI6Mn0%3D%7C3000%7C%7C%7C&sdata=4YMf1jKxopYmPZ8RaftD7bziOo1wMQ0Xs9T9uFbJ34s%3D&reserved=0)  When completing the checklist, please use section and paragraph numbers, rather than page numbers. | We have included the STROBE checklist in Supplementary item 1 and included the statement as advised. | 268 |
| 10 | Title | Please revise your title according to PLOS Medicine's style. Please note that country and study design should be in the title and please place the study design ("A randomized controlled trial," "A retrospective study," "A modelling study," etc.) in the subtitle (ie, after a colon), | We have changed the title to ‘Diagnosis and Management of Endometrial Hyperplasia: A UK National Audit of Adherence to National Guidance’. |  |
| 11 | Data availability statement | The Data Availability Statement (DAS) requires revision. For each data source used in your study: a) If the data are freely or publicly available, note this and state the location of the data: within the paper, in Supporting Information files, or in a public repository (include the DOI or accession number).  b) If the data are owned by a third party but freely available upon request, please note this and state the owner of the data set and contact information for data requests (web or email address). Note that a study author cannot be the contact person for the data.  c) If the data are not freely available, please describe briefly the ethical, legal, or contractual restriction that prevents you from sharing it. Please also include an appropriate contact (web or email address) for inquiries (again, this cannot be a study author).  In your case, it seems option c is applicable, but we require a bit more information (e.g reason for the data restriction and please not that note that a study author cannot be the contact person to request access to the data. | We have updated the statement on data availability as advised, in the submission metadata and have identified a third party to be the point of contact to request access to the data. We have detailed this in the submission metadata. |  |
| 12 | Abstract | Please structure your abstract using the PLOS Medicine headings (Background, Methods and Findings, Conclusions). | Thank you, we have made these changes. |  |
| 13 | Abstract background | Provide the context of why the study is important. The final sentence should clearly state the study question. | We apologise that the context provided was not clear, we have better emphasised that there is a high risk of patients having concurrent, undiagnosed endometrial cancer and that adherence to current UK guidance is unknown but warrants audit to identify opportunities for quality improvement. | 243-264 |
| 14 | Abstract Methods and Findings | Please include the study design, population and setting, number of participants, years during which the study took place, length of follow up, and main outcome measures. | Thank you, this has been edited to include these details. |  |
| 15 | Abstract Methods and Findings | Please quantify the main results (with 95% CIs and p values). Suggest reporting statistical information for clarity in the following format: ‘x’; (95% CI [‘y’,’ z’] p value) (e.g  Prior to national guidance, 9% (95% CI [6%,15%] p value) received …..). For p values, please report as p<0.001 and where higher as 'p=0.002'. | Thank you, throughout the abstract and main manuscript we have changed the format of all presented results. |  |
| 16 | Abstract Methods and Findings | In the last sentence of the Abstract Methods and Findings section, please describe the main limitation(s) of the study's methodology. | We have better incorporated the limitations into a section under ‘Strengths and limitations’. | 771 |
| 17 | Author summary | In the final bullet point of ‘What Do These Findings Mean?’ Please include the main limitations of the study in non-technical language. | Thank you, we have added a statement in this section to give an overview of our limitations. | 771 |
| 18 | Introduction | Please expand your introduction, address past research, context of study, and explain the need for and potential importance of your study. | Thank you for your feedback. We have expanded the introduction with contextualisation of the care of women with EH, evidence of potential variation, and the relevance and importance of this study as discussed above. |  |
| 19 | Methods | Population: please include the number of participants | We have added the total number of participants to the population section of the methods. |  |
| 20 | Discussion | Please present the Discussion as follows: a short, clear summary of the article's findings; what the study adds to existing research and where and why the results may differ from previous research; strengths and limitations of the study; implications and next steps for research, clinical practice, and/or public policy; followed by a one-paragraph conclusion. | The discussion is now presented under the following subheadings:  Principal findings, results in the context of what is known, strengths and limitations, implications for clinical practice, future directions, conclusion. | 636 |
| 21 | Discussion | Please consider expanding discussion on global endometrial hyperplasia care in comparison to the UK care. | We have included reference to the recommendations on care in other settings where guidance is available. Unfortunately, there is limited description of care in other settings. | 684-734 |
| 22 | Acknowledgements  Declarations | Please remove all statements apart from acknowledgements, author contributions and abbreviations from the main manuscript and include these only in the relevant parts of the manuscript submission form. Funding, competing interest, and data availability will be compiled as metadata. | Thank you – we have updated the manuscript accordingly (working group authors removed from main text). |  |
| 23 | References | Please use the "Vancouver" style for reference formatting, and see our website for other reference guidelines [https://journals.plos.org/plosmedicine/s/submission-guidelines#loc-references](https://eur01.safelinks.protection.outlook.com/?url=https%3A%2F%2Fjournals.plos.org%2Fplosmedicine%2Fs%2Fsubmission-guidelines%23loc-references&data=05%7C01%7C%7Cfee979e3ac594d175ebe08dbcbcbf1f8%7C09bacfbd47ef446592653546f2eaf6bc%7C0%7C0%7C638327849040446571%7CUnknown%7CTWFpbGZsb3d8eyJWIjoiMC4wLjAwMDAiLCJQIjoiV2luMzIiLCJBTiI6Ik1haWwiLCJXVCI6Mn0%3D%7C3000%7C%7C%7C&sdata=lceVgPRjOXN5793BgrVhxU%2BahS9eaJjHKOCoJozFhj4%3D&reserved=0)  Please ensure that any references to online-only sources include a date of accession (e.g see reference 10) | We have included the dates of accession where omitted. |  |
|  |  |  |  |  |
| Comments from the reviewers: | | | |  |
| Reviewer #1: | | | |  |
| No. | Area | Comment | Response |  |
| 24 | General | This manuscript is an interesting and ambitious audit of endometrial hyperplasia (EH) treatment over a 9-year period in the UK. The results show a generally high level of guideline-consistent treatment yet identify specific areas where adherence to guidelines could be improved. The methods are strong and the analysis & results are presented in a clear and understandable way. Below are a few questions & suggestions. | Thank you for taking the time to review and engage with our work and for your helpful feedback. |  |
| 25 | Introduction | Is it possible to summarize what the generally accepted treatment patterns or tendencies were in the UK before the introduction of the GTG in 2016? Or to provide any data on how consistent, or varying, treatment for NEH and AEH were before the GTG was introduced? | Thank you, we have added detail regarding the treatment patterns of EH prior to the introduction of the GTG guidance, although the understanding of care was limited. |  |
| 26 | Materials & Methods | On p. 5 the population section doesn't include numbers; later these are provided, in results, but as I read the population section I wished those numbers had been provided there. | Thank you, we have inserted this into the population of the methods section. |  |
| 27 | Study design | Under Study Design, the collection of data from "physical or electronic records" is mentioned briefly. More details about that step could provide information readers could use to assess the quality of the data collection. | We have expanded this section to detail the different information sources accessible to the data collector. |  |
| 28 | Study design | Also, this section mentions data collection at the level of the individual patient, whereas the Figure 1 refers to "units", which presumably are clinical units. Adding patient-level data to the figure could provide broader context to which patients were included. | That’s correct – we collected patient-level data clustered at the level of the hospital. We have referred to ‘patients’ and ‘hospitals’ as opposed to ‘observations’ and ‘units’ in Figure 1 as perhaps this was unclear. Apologies if we have not understood this feedback. |  |
| 29 | Outcomes | Under outcomes, is there a specific rationale for choosing 42 days as the time interval for first-line management? Is that based on any recommendation or guidance? | We selected 42 days as the threshold to allow time after the initial biopsy for the histology (whether requested as routine or urgent) to be processed and reported, for the requesting clinician to action the result and for the plan to be documented in the patient notes. There are no national guidelines for turnaround times for routine histology but we would not envisage routine endometrial samples to take longer.  Separately, some patients were reviewed in an outpatient clinic with an abnormal ultrasound scan and underwent a blind endometrial biopsy in clinic but were simultaneously booked for a diagnostic hysteroscopy. These hysteroscopies did not represent the first-line management of a histologically-diagnosed endometrial hyperplasia, so we were better able to capture the first-line management by using a broader period of time.  We have better reflected this in the outcomes section. | 319-324 |
| 30 | Statistical analyses | The statistical analysis section mentions relative risks; the model or methods for generating those should be added. Because those metrics appear to be before-vs-after comparisons--i.e., comparing all 2012-2016 patients with all 2017-2020 patients--are those metrics in fact closer to odds ratios, rather than relative risks?  That is, the result describes the odds of a given treatment among a clinical subgroup before the GTG vs. after the GTG. | As the outcomes/process measures were common, we estimated relative risks (now termed ‘rate ratios’) only for ease of interpretability by a clinical and lay audience. We hope that the updated terminology of rate ratios makes this clearer. |  |
| 31 | Results | This comment is strictly a personal preference:  the results are thorough and clear to navigate, but the tables are underwhelming. The nature of these comparisons--percentages before vs. after; changes in percent treated before 2016 vs. after--make them well-suited to presentation of data in figures, rather than just tables. | Thank you for your suggestion. As we have now included the rate ratios and p-values in the tables, we have not included a graphical representation either in the tables or elsewhere. We hope that the side-by-side rate-ratios aids the interpretation. |  |
| 32 | Discussion | Overall, this section accurately summarizes the results and addresses important contextual factors. Some of the phrases in the first paragraph struck me as perhaps moving beyond what the data showed or could support. Some examples:  The first sentence includes "were more likely to avoid hysterectomy"; avoid implies hysterectomy would not be justified, but in some cases it might be. | We have rephrased this sentence about avoiding hysterectomy to state that women with NEH were more likely to receive intrauterine progestogen and less likely to undergo hysterectomy. | 645 |
| 33 | Discussion | In the middle of the first sentence, "Nevertheless, there is a need for the follow-up of women with either NEH or AEH to improve" seems incomplete; in other sections of the Discussion, specific areas for improvement are provided, and those sections are much more convincing. | Thank you - this sentence has been joined with the subsequent sentence and hopefully now makes more sense. | 650 |
| 34 | Discussion | The final sentence mentions the need for urgent follow-up of patients whose care might have been interrupted by Covid in 2020; at the time of this review, it's already 3 years after those 2020 visits. | We have rephrased this sentence to state that clinicians should ensure standard care has resumed for the patients affected by the COVID pandemic. | 653-656 |
| 35 | Discussion | In the Research implications, the text appears to put most of the responsibility for improved follow-up on patients:  e.g., patients should receive better communication or pamphlets. The assumption appears to then be that this would empower patients to express their preferences, but this whole section seems to under-appreciate or recognize the role that providers and the clinical care system have in influencing patients' decisions. This speaks to a broader area that is not really covered in the manuscript:  the area of implementation science. Some comment on what steps the UK gynecological oncology community made to disseminate the GTG, train providers, and provide continuing monitoring & education for achieving optimal first-line and follow-up care could be useful in this manuscript. | Although we have characterised the follow-up pattern in as much detail as possible, the clinical documentation did not always reveal the exact reason for loss to follow-up. We reference that patients did not attend only because this was one of few specific reasons that were documented. We wished to emphasise that the clinicians could ensure high-quality counselling on the risks and benefits of follow-up and treatment and support their counselling with patient-centred literature or decision aids (given that there could be an under-appreciation of the risks of both NEH or AEH and therefore poor communication of the true risk to a patient). We have developed this aspect of the discussion accordingly, emphasising that clinicians must offer all women with NEH treatment with follow-up or follow-up if the patient elects for conservative management and all women with AEH hysterectomy or medical management if hysterectomy is inappropriate or declined. We have led with the importance of evidence based care from clinicians, and amended this section to focus on supporting counselling and communication, with a focus on healthcare systems rather than patients. Thank you for highlighting this important point. | 813-818 |
|  |  |  |  |  |
| Reviewer #2: | | | |  |
| No. | Area | Comment | Response |  |
| 36 | General | Thanks for inviting me to review this manuscript, assessing the real-world practice for endometrial hyperplasia in UK. A quasi-experimental method was used to assess the possible causality of practice guideline recommendation on the 1st line of treatment. I have the following comments that the investigators may develop further if it improves the study quality. | Thank you for taking the time to review our paper and for your constructive comments. |  |
| 37 | Study design | 1 Selection bias. I saw that large number of patients were excluded due to lack of minimum 2-year follow-up (>400 patients). This was to assess the 2-year time point hysterectomy rate in the second step of analysis. The investigator did not provide the data-driven rationale to use 2-year cut-point. | The exclusion of patients regarding the two-year status measure only affected those whose first biopsy was at the lattermost period of the study; that is, these women were ineligible for the two-year measure as we did not know their outcome rather than being excluded for ‘missing’ data. We appreciate that this may have been unclear and have now clarified this point.  Conversely, if a patient was lost to follow-up then we described the type of loss to follow-up as a measure of interest based on the documentation or absence of documentation. As all patients should have received some form of follow-up within two years across the study, we regard women with no documentation as lost to follow-up.  The selection of the two-year threshold was primarily based on the recommendation for hysterectomy for non-atypical EH if there is no response by 12 months. This meant that a two-year time period allowed for women who trialled initial medical management to return for biopsy, for the biopsy result to become known, and then for hysterectomy to be performed. We have added detail on the selection of the two year follow up period. | 354-58 |
| 38 | Study design | Majority of EH respond to progestin therapy within 1-year time period. So, among the 400 patients who were excluded due to <2 years of follow-up after the first-line treatment, it is likely that there were many patients already regressed and may not have needed hysterectomy. | Yes, many of the women who had their diagnosis of EH in these latter 6 months of the study period did have follow-up biopsies which showed regression. We did not deem these women eligible for inclusion in the two-year analysis because some women in this latter time period had not yet received follow-up and likely would have by the end of the two-year interval and this may have introduced bias in favour of the pre-guidance group. We wished to ensure that all women who were compared in either group had the same potential period for follow-up. |  |
| 39 | Results | To minimize this selection bias, the investigators might want to consider time-dependent analysis with competing risk. For instance, event of interest (hysterectomy) can be scored as 1 occurred during the follow-up. Then, competing event can be scored as 2 (e.g, death) occurred during the follow-up. Patient who did not have these vent at last follow-up can be scored as 0. Difference-in-Difference (DoD) analysis will be considered to compare pre- and post-guideline implementation periods. | We considered time-to-event analyses but the follow-up period was very short for the majority of women and follow-up data are coarse, with a long tail of very few women having regular follow-up. |  |
| 40 | Results | 2 Information on temporal trend for NEH / AEH is missing but this will provide important insights. Is AEH increasing over time? | Unfortunately, we were unable to comment on the incidence of EH over time due to the audit case identification process and our inability to define the denominator. Although there was an increase in cases in the post-guidance period, we cannot determine what proportion of the increase in cases may be true and what may represent a differential identification of cases (potentially due to either increased contact with secondary healthcare or due to improved quality of documentation systems ie. electronic documentation). We have added discussion of this limitation. | 785-787 |
| 41 | Results | 3 Subsequent hysterectomy rates differ between IUD vs systemic progestin. This can be factored in the analysis. | Thank you for your suggestion. We modelled the outcomes with respect to change over time in the first instance as this matched our main descriptive audit objectives.  We have now additionally compared the rates of histological resolution at two years among women who received medical management as a post-hoc exploratory analysis which we have detailed in the methods under post-hoc analyses and have reported in-text. We chose to compare histological resolution because the decision to undertake hysterectomy or not may also be associated with the selection of progestogen route. We adjusted for factors we believe may influence selection of route (age, BMI, parity, subfertility). | 411-414 |
| 42 | Abstract | 4 Abstract conclusion second sentence. This sentence is unclear and might benefit of clarification. For example, there is no description for occult cancer in abstract result section. | Thank you, we apologise that this was not clear. We have reworded the sentence on endometrial cancer in the abstract to emphasise that 37% of those with AEH who underwent first-line hysterectomy had endometrial cancer not diagnosed by the initial biopsy. We have replaced the word ‘occult’ with ‘co-existent’ in the conclusion to hopefully make the significance of this finding clearer. | 140 |
|  |  |  |  |  |
| Reviewer #3: Alex McConnachie, Statistical Review | | | |  |
| 43 | General | Henderson et al present the results of a nationwide audit of the management of endometrial hyperplasia in the UK, covering a 9-year period spanning the introduction of new guidance. This review considers the use of statistics in the paper.  Overall, if thought the paper was nicely written, giving clear explanations of where the data came from, and what the exposures and outcomes were. Saying that, I found the paragraph at the top of page 7 to be a little confusing (I think lines 320-326, but the numbering on my copy of the paper is not fully visible). Hopefully this makes sense to a clinical reader. The statistical methods seem reasonable, using basic descriptive methods, accounting for clustering at the hospital level when calculating confidence intervals. | Thank you for your positive feedback and taking the time to read the manuscript and comment on our work.  Regarding the paragraph that was lines 320-326 we have added further clarification on the selection of the 42-day window. | 323-324 |
| 44 | Study design | There is no explanation of the sample size for the audit. For an audit, I would not necessarily expect a formal power calculation, but was there a rationale for studying this number of hospitals (are these the only hospitals in the UK that treat these patients, or just the ones that responded?) or for this particular time frame (why 9 years, and not 10)? | Thank you, we agree that these areas need further clarification.  For this project, UKARCOG regional representatives contacted clinicians who worked at any UK based gynaecology unit to recruit them as local data collectors. Not all units responded, the included units are specified in the appendix.  We chose to the time period of 1/1/12-31/06/20 to capture the standard practice in these units prior to and following the guideline introduction in early 2016. With respect to the description of contemporary clinical practice, the time period of 2016-2020 was fixed. We then selected the earlier time frame to roughly match the period and according to National Health Service guidance on the retention of medical records (Records Management Code of Practice, NHS England 2023) that medical records are retained for 8 years from the last treatment, given that some medical records would be handheld notes.  Subsequently, as we saw that practice changed at the onset of the Covid-19 pandemic, we have described cases from 2020 separately and we plan to conduct further work and data collection to understand these changes.  We have better described the selection of the time period in the methods. | 282-302 |
| 45 | Statistical analyses | The methods section says that relative risks are calculated, and that they "modelled first-line hysterectomy on patient characteristics". A little more information could be given here - what kind of models are we talking about? If the results are presented as relative risks, was it a Poisson model? How did the model account for hospital clustering? Were any covariates included in the models? | Apologies for this omission. The relative risks were estimated using multilevel Poisson regression with random intercepts for hospital unit. We have added this detail in the methods. We have also included our rationale for the specification of this model. | 391-424 |
| 46 | Statistical analyses/General | Incidentally, when looking at an outcome that is not necessarily an adverse event, such as receiving a particular kind of treatment, I always find the term "relative risk" to be out of place. Is "rate ratio" an acceptable alternative? | Thank you for highlighting this oddity. We agree that “rate ratio” is more suitable than “relative risk” and have changed this terminology throughout the manuscript. |  |
| 47 | Statistical analyses/General | The reporting of results in the main text of the paper is fine, except for the tendency to repeat things. For example, the bottom line of page 9 includes "31% (216/ 701, 31% [95% CI 26-36%])" - the "31%" is repeated. In other places, it is the numerator that is repeated, for example on page 11 I saw "25 (25/26, 96%)". None of this is wrong, just a little jarring. | We agree and have removed any duplicated reporting of results throughout the manuscript. |  |
| 48 | Results | In Table 1, would it be of value to show NEH and AEH separately, pre- and post-guidance? The total columns could be dropped to make space. Also, for age and BMI, were these only recorded in categories, or are the continuous values available? If so, why not report as mean and standard deviation, or as median and quartiles? It might make the differences (if any) easier to see. In tables 2-4, I think the columns would be better ordered differently. If the main exposure of interest in the introduction of the guidance, then I would have two columns for NEH (pre- and post-guidance) side by side, followed by the same for AEH. That way it is easier to compare the different time periods. | Thank you for this suggestion. We have amended Table 1 accordingly, including the description of age using mean years (although we did capture BMI categorically). We have reconfigured Tables 2-4 accordingly for easier comparison as suggested. |  |
| 49 | Results | Also in tables 2-4, I would try to include the RR estimates and confidence intervals from Supplementary Table 2. If space is an issue, the p-values could be dropped. I think it is important to see the data presented by time period alongside the estimated associations, to get the full picture. | We have included the rate ratios from the supplementary table into Tables 2-4. We have changed the tables into a portrait layout to allow space to do so. Some of the rate ratios in the previous supplementary table related to specific auditable points (like ‘TLH BSO if postmenopausal’) but for consistency we have reported the additional, previously omitted, rate ratios. |  |
| 50 | Results | Supplementary Table 3 looks at first-line hysterectomy in relation to patient characteristics. It shows percentages with different characteristics, according to whether first line management was hysterectomy or not. For me, this is the wrong way round. Here, the outcome is hysterectomy (or not), so rather than showing column percentages (i.e. of those with hysterectomy, how many were in each age group?) it should show row percentages (in each age group, how many had hysterectomy?). | In this supplementary table we considered patients from both time periods combined because we did not suspect that the determinants of hysterectomy would necessarily differ over time; however, we agree that it would be beneficial to consider the potential reasons women did not undergo hysterectomy for AEH more comprehensively. |  |
| 51 | Results | If presented in this way, the no hysterectomy column would not need to be shown. E.g., for age <40, the table could show that there were 130 women with AEH, and 27 (21%) received first line hysterectomy. You could then have a second column showing the RR estimates and confidence intervals for the associations between each patient characteristic and the likelihood of receiving first line hysterectomy. This would complement Figure 5 in the paper. Incidentally, why are diabetes and hypertension not shown in Supp Table 3 (or is "blood glucose disorder" the same as diabetes?). | We have now separated the outcome by time period and changed the column percentages to row percentages and included the univariable and multivariable (for all potential explanatory variables/risk-factors combined). The latter time period therefore better describes the more contemporaneous post-guidance practice. |  |
| 52 | Results | When looking at these associations, you could look at each patient characteristic individually, or you could try to fit a multivariable model, considering all predictors simultaneously. | As above |  |
| 53 | Results | Finally, and I may be going to far at this point, the table could also be split into pre- and post-guidance columns. You could then ask whether the factors predictive of first line hysterectomy are the same post-guidance as they were pre-guidance. Ideally, this would be done by fitting regression models with interaction terms, but there is probably not much power in such analyses. Even so, to look at these associations in the two time periods may be interesting, even if the same factors predict hysterectomy in both periods. | In an additional exploratory analysis, we have tested for statistical interaction between the risk-factors and time period for the outcome of hysterectomy among women with AEH. As we did not identify any significant interaction terms and as we did not appreciate any differences when comparing predicted probabilities from the model with and without interaction terms, we have presented the risk-factor model without interaction terms. We have added this exploratory analysis to the methods. |  |
